# Factors Driving Citizen Engagement With Government TikTok Accounts During the COVID-19 Pandemic: Model Development and Analysis

**DOI:** 10.2196/21463

**Published:** 2021-02-04

**Authors:** Qiang Chen, Chen Min, Wei Zhang, Xiaoyue Ma, Richard Evans

**Affiliations:** 1 School of Journalism and New Media Xi’an Jiaotong University Xi'an China; 2 Department of Media and Communication City University of Hong Kong Hong Kong China; 3 College of Public Administration Huazhong University of Science and Technology Wuhan China; 4 School of Medicine and Health Management Huazhong University of Science and Technology Wuhan China; 5 College of Engineering, Design and Physical Sciences Brunel University London London United Kingdom

**Keywords:** government social media, citizen engagement, public health crisis, TikTok, emotion valence, dialogic loop, COVID-19

## Abstract

**Background:**

During the COVID-19 pandemic, growth in citizen engagement with social media platforms has enabled public health departments to accelerate and improve health information dissemination, developing transparency and trust between governments and citizens. In light of these benefits, it is imperative to learn the antecedents and underlying mechanisms for this to maintain and enhance engagement.

**Objective:**

The aim of this study is to determine the factors and influencing mechanisms related to citizen engagement with the TikTok account of the National Health Commission of China during the COVID-19 pandemic.

**Methods:**

Using a web crawler, 355 short videos were collected from the Healthy China account on TikTok (with more than 3 million followers throughout China), covering the period from January 21, 2020, to April 25, 2020. The title and video length, as well as the number of likes, shares, and comments were collected for each video. After classifying them using content analysis, a series of negative binomial regression analyses were completed.

**Results:**

Among the 355 videos, 154 (43.4%) related to guidance for clinicians, patients, and ordinary citizens, followed by information concerning the government’s handling of the pandemic (n=100, 28.2%), the latest news about COVID-19 (n=61, 17.2%), and appreciation toward frontline emergency services (n=40, 11.3%). Video length, titles, dialogic loop, and content type all influenced the level of citizen engagement. Specifically, video length was negatively associated with the number of likes (incidence rate ratio [IRR]=0.19, *P*<.001) and comments (IRR=0.39, *P*<.001). Title length was positively related to the number of shares (IRR=24.25, *P*=.01), likes (IRR=8.50, *P*=.03), and comments (IRR=7.85, *P*=.02). Dialogic loop negatively predicted the number of shares (IRR=0.56, *P*=.03). In comparison to appreciative information, information about the government’s handling of the situation (IRR=5.16, *P*<.001) and guidelines information (IRR=7.31, *P*<.001) were positively correlated with the number of shares, while the latest news was negatively related to the number of likes received (IRR=0.46, *P*=.004). More importantly, the relationship between predictors and citizen engagement was moderated by the emotional valence of video titles. Longer videos with positive titles received a higher number of likes (IRR=21.72, *P*=.04) and comments (IRR=10.14, *P*=.047). Furthermore, for short videos related to government handling of the pandemic (IRR=14.48, P=.04) and guidance for stakeholders (IRR=7.59, *P*=.04), positive titles received a greater number of shares. Videos related to the latest news (IRR=66.69, *P*=.04) received more likes if the video title displayed higher levels of positive emotion.

**Conclusions:**

During the COVID-19 pandemic, videos were frequently published on government social media platforms. Video length, title, dialogic loop, and content type significantly influenced the level of citizen engagement. These relationships were moderated by the emotional valence of the video’s title. Our findings have implications for maintaining and enhancing citizen engagement via government social media.

## Introduction

### Background

Citizen engagement on social media during times of public health crises, such as that experienced during the COVID-19 pandemic, provides governments with a valuable tool for communicating with and understanding the concerns and priorities of their citizens [1]. Citizen engagement highlights the active role citizens play in public affairs, including public communication, public consultation, and public participation, which have the potential to influence government decision making [2]. With citizen engagement, governments can solicit public concerns and respond accordingly, improving the quality and efficiency of public services delivered [3]. Due to the popularity and communicative nature of social media, it has been employed widely by governments during public health crises, creating new opportunities for citizens to voluntarily participate in government activities [4-8]. However, the practice of engaging citizens is relatively underdeveloped. Strategies for citizen engagement via social media platforms have not been adequately deployed, although the importance of citizen engagement is widely acknowledged by government agencies. The use of social media by governments during public crises is hampered by both internal and external factors, such as insufficient resources, the digital divide, ethical considerations, and accountability [9,10]. Government agencies thus prefer to broadcast information and improve disease surveillance through social media, rather than initiate public conversation and engagement [11-13]. Considering its benefits, it is imperative to better understand the antecedents and underlying mechanisms of citizen engagement with government social media during public health emergencies to maintain and enhance citizen engagement.

Although some studies have examined the relationship between government agencies’ use of social media and citizen engagement during public health crises, there are several obvious gaps that deserve further investigation. First, existing studies have paid extensive attention to more traditional social media, including Sina Weibo [1,8], Twitter [4,14], and Facebook [7]. Chen and colleagues [14] identified the tweeting patterns of the Centers for Disease Control and Prevention during different phases of the Zika epidemic. However, their studies focused on text-dominated social media exclusively, with little attention paid to emerging social media platforms. For example, TikTok, which launched in 2016, is a video-sharing platform that allows users to create, upload, repost, and write comments on short videos, ranging from a few seconds to minutes. Since its creation, the popularity of TikTok has increased rapidly, with more than 800 million active users in 2020 [15]. During the COVID-19 pandemic, TikTok became an indispensable source of information and communication channel for the general public and between government organizations and citizens [16]. Second, extant studies have predominantly explored possible influencing factors through the lens of media type, government type, content type, media richness, message style, and interactive features, with limited effort paid to specific influencing mechanisms [1,4,8]. Guidry et al [4] explored the Ebola-related social media posts published by three major health organizations on Twitter and Instagram, and found that messages with solutions, visual imagery, and acknowledgment of public concerns are the most effective risk communication strategies. By investigating the People’s Daily Sina Weibo account during COVID-19, Ngai et al [8] found that disease prevention content posts in a narrative style generated more public engagement, and further revealed an interaction effect between content and style.

This study aims to address the above gaps by exploring the factors that drove citizen engagement through the TikTok account of the National Health Commission of China (NHCC) during the COVID-19 pandemic, in the context of China. Chinese governments, across different levels, have now started to realize the potential of TikTok for public participation, leveraging the platform for routine administration tasks. According to the latest report by the China Internet Network Information Center (CNNIC), the number of verified government TikTok accounts is 25,313, implying wide employment in the Chinese public sector [17]. In this study, we develop a conceptual model, including video length, title length, dialogic loop, and content type, to empirically examine the driving factors of citizen engagement with TikTok accounts operated by Chinese government agencies. Citizen engagement through the official TikTok account of the NHCC consists of three dimensions: comments, likes, and shares [18]. For each video uploaded to TikTok, data on its number of comments, likes, and shares are publicly available. Importantly, we take the emotional valence variable as a moderator to unravel potential influencing mechanisms.

### Research Hypotheses and Conceptual Model

**Figure 1:**
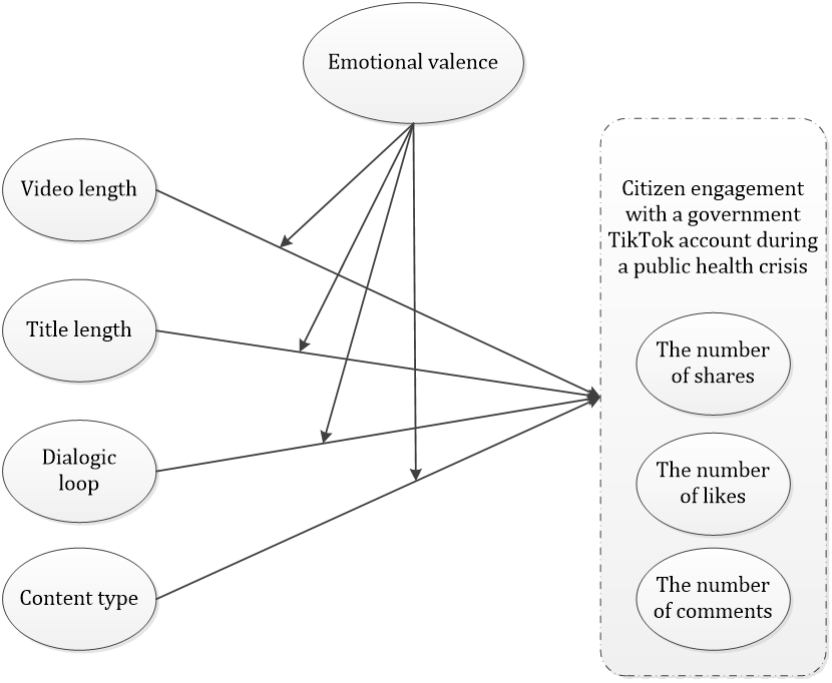
The research model of the study.

Based on the characteristics of TikTok videos, our study built a moderated model that unraveled the moderating role of emotional valence among the differentiated effects of video length, title length, dialogic loop, and content type on the three dimensions of citizen engagement, namely the number of shares, likes, and comments (Figure 1).

Video length refers to the duration of a video. Although several studies have examined the length of popular videos, few have investigated the impact of video length on citizen engagement on TikTok. For example, Zhu et al [19] analyzed the 100 most-liked videos on TikTok published by 31 provincial Health Committees in China, and found that 98% of them lasted under 60 seconds. Our study suggests that video length is negatively associated with the number of likes, shares, and comments received from the public. Shorter videos usually have better content because they require detailed planning to explain the content concisely [20]. Guo et al [20] analyzed a big data set comprising 6.9 million Massive Open Online Course (MOOC) videos and confirmed that shorter videos had a perceived higher quality, thus leading to increased engagement. In addition, shorter videos align better with the information consumption habits of social media users. Users usually watch TikTok videos on mobile devices during fragmented periods. If a video is too long, it will not only consume more mobile data and battery power, but also fail to meet the demands of individuals’ allotted time.

Therefore, hypothesis 1 is as follows: video length negatively affects the three dimensions of citizen engagement via the official TikTok account of the NHCC, as measured by the number of likes, shares, and comments.

Title length refers to the total number of words summarizing the video’s content in the title of an uploaded video. During times of crisis, citizens are usually motived by utility, craving information that relieves anxiety and eases panic [21]. Longer titles typically contain richer information, promoting a better understanding of the video, thereby increasing citizen engagement [21,22]. For example, Xu and Zhang [22] examined 13,322 tweets related to Malaysia Airlines Flight 370 and found that the more words a tweet had, the more likely it was to be retweeted. Specific to video-sharing platforms, Halvey and Keane [23] analyzed 4.25 million YouTube videos and identified that a longer video title increased the number of video views. We argue that the longer the title length, the more likely citizens are to share, comment, and like COVID-19–related TikTok videos.

Therefore, hypothesis 2 is as follows: title length positively affects the three dimensions of citizen engagement via the official TikTok account of the NHCC, as measured by the number of likes, shares, and comments.

Dialogic loop emphasizes the importance of providing an interactive feedback loop, facilitating mutual communication between organizations and citizens [1]. Organizations allow the general public to directly ask questions, post comments, make suggestions, and provide feedback on relevant issues pertaining to the organization [24]. Dialogic loop is beneficial for promoting mutual conversation through question posing and answering [1]. However, few studies have investigated the effect of dialogic loop on citizen engagement via social media. Gálvez-Rodríguez et al [25] analyzed 137 Latin American local governments’ Facebook accounts and revealed that dialogic loop can increase the level of citizen engagement (weighed calculation based on the number of likes, comments, shares, and posts). Specific to public health crises, Chen et al [1] studied the official Sina Weibo account of the NHCC and verified that dialogic loop positively influences citizen engagement (sum of number of likes, comments, and shares).

Therefore, hypothesis 3 is as follows: dialogic loop positively affects the three dimensions of citizen engagement via the official TikTok account of the NHCC, as measured by the number of likes, shares, and comments.

Extant studies into health communication have identified that different content types have varying effects on citizens’ social media engagement behaviors. According to the Use and Gratification Theory, the degree to which media content gratifies individuals’ differential needs directly influences their media selection and usage behavior [26]. Analyzing 203,191 COVID-19–related posts on Sina Weibo, Wang et al [27] concluded that posts on the domestic epidemic were retweeted the most, while posts about quarantine and investigations received the most likes. Short videos related to COVID-19, posted by the official TikTok account of the NHCC during the pandemic, will satisfy public demands to different degrees, thus promoting citizen engagement at different levels. Research that analyzed 4221 tweets posted by the health departments of 39 states in the United States showed that acknowledging the events of other organizations and expressing gratitude and providing recognition improved citizen engagement [28]. Park et al [29] examined 1583 tweets posted by three American health organizations and found that personal health–related information and actions received more shares and likes. In relation to public health crises, Chen et al [1] determined that posts about the latest news and government disposition facilitated citizen engagement via government Sina Weibo accounts, while posts providing guidance for stakeholders had no effect.

Therefore, hypothesis 4 is as follows: content type has significant differential effects on the three dimensions of citizen engagement via the official TikTok account of the NHCC, as measured by the number of likes, shares, and comments.

Emotional valence refers to the positive and negative feelings triggered during an individual’s consumption of information [1,18]. Emotional expression in social media content can attract public attention, promote dialogue, and drive engaged feedback [18]. Emotions possess the function of physiological arousal, which can contribute to information-sharing behavior [30]. High emotional traits can trigger the viral spread of social media content [22]. Citizens regulate their emotional status through actions such as reposting, liking, and commenting when receiving emotional information via social media [1]. Empirical evidence indicates that emotional posts often promote citizens’ social media engagement [18,31,32]. However, few studies have investigated the moderating effect of emotional valence [33]. Tang et al [34] studied the official Sina Weibo accounts of 30 provincial police departments in China and identified that emotional valence moderated the influence of content type on reposting behavior. Child-friendly content with positive emotion increased reposting the most. Current research has also confirmed that emotional valence moderates the effects of dialogic loop, media richness, and content type on citizen engagement via government Sina Weibo accounts during public health crises [1].

Therefore, hypothesis 5 is as follows: emotional valence will moderate the effects of video length, title length, dialogic loop, and content type on the three dimensions of citizen engagement via the official TikTok account of the NHCC, as measured by the number of likes, shares, and comments.

## Methods

### Data Collection

For this study, we collected data from the official TikTok account of the NHCC, “Healthy China.” The official account was created on May 4, 2018, to help more widely disseminate health-related information. By May 12, 2020, the account had posted 576 short videos, had 3 million followers, and had received more than 8 million likes. Since the outbreak of COVID-19 in December 2019, the NHCC has actively uploaded relevant short videos. We used a web crawler tool to capture all videos uploaded to the official account from January 21, 2020, to April 25, 2020. In total, 364 videos were obtained. After manual checking, 355 were found to be related to the COVID-19 crisis. In addition, data about the video’s length, title text, number of likes, number of shares, and number of comments were collected.

### Operationalization of Variables

Citizen engagement through the official TikTok account of the NHCC includes three dimensions: sharing, liking, and commenting behaviors [18]. We captured this objective data using web crawlers.

Video length is the duration of the video. The length of all 355 videos was captured from the “Healthy China” account using a web crawler.

Title length relates to the number of words included in a video’s title. This study first collected the complete texts of 355 video titles using web crawlers, and then automatically counted the number of words in each text using Microsoft Excel (Microsoft Corp).

Dialogic loop is measured by two indicators: posing and answering questions [35]. If a question or answer appears in a short video title, it is marked as 1, otherwise it is marked as 0. The dialogic loop score was the sum of the scores of the two indicators.

Content type includes 4 categories, namely the following: the latest news about the COVID-19 crisis, the government’s handling of the pandemic, appreciation for frontline emergency services, and guidance for stakeholders [1]; the codebook in Table 1 contains examples.

**Table 1:** Codebook examples of content categories.

Categories	Example posts
The latest news about the COVID-19 crisis	[Authoritative Release] The latest situation of COVID-19 crisis as of 24:00 on April 11#Action to defeat COVID-19
The government’s handling of the crisis	Covid-19 prevention, control and medical treatment work in Hubei, from the press conference of the State Council Information Office. #Action to defeat COVID-19
Appreciation of frontline emergency services	Thank you, Sichuan Provincial Medical Aid Team, tribute heroes triumphantly! #Action to defeat COVID-19 #Warm doctor moment
Guidance for stakeholders	Parents look out! How can children prevent COVID-19? #Action to defeat COVID-19

### Emotional Valence

We measured this variable by calculating the emotional valence of each short video’s title based on a sentiment lexicon and Python (Python Software Foundation). Initially, title information was split into three categories (sentimental words, negative words, and degree adverbs) using the Jieba database. Upon word separation, the words were annotated using BosonNLP, while machine learning enabled word processing to assign values for sentimental words. By combining the review of the rest of the words with preassigned values, the emotional value for each title was determined based on a weighted approach. Each title was assigned a value ranging from 0 to 1, with 0.5 being neutral emotion. The closer the value was to 0, the more negative the emotion was considered, and vice versa.

### Intercoder Reliability and Data Analysis

This study obtained the analysis data of dialogic loop and content type by manually coding the content of the video titles. We employed two graduate students to carry out the coding of the 355 short videos. Before beginning, the students were provided with operation training and an explanation of the coding scheme to ensure a good coding standard. To examine the interreliability, 30% of the sample was randomly selected, and 106 short videos were precoded. Both coders worked independently, and the results are as follows: the κ values for the content categories, “posting a question,” and “responding to a question” were 0.918, 0.941, and 0.795, respectively. This indicated that the interreliabilities were high enough and acceptable.

Our dependent variables are all count measures that exhibit overdispersion (shares: mean 86.94 [SD 492.71], skewness=11.36, kurtosis=140.27; likes: mean 1464.22 [SD 4772.29], skewness=6.94, kurtosis=57.85; comments: mean 7.57 [SD 13.22], skewness=4.10, kurtosis=26.07). A significant number of short videos rarely received shares, likes, or comments, while others received a high number in comparison. To deal with this overdispersed count data, we modelled the number of shares, likes, and comments using negative binomial regression. We first estimated the impact of video length, title length, dialogic loop, and content type on different types of citizen engagement. We then explored whether the impacts were contingent upon the emotional valence of each video’s title. All analyses were conducted using STATA (Version 15.0; StataCorp LLC).

## Results

### Descriptive Analysis

**Figure 2:**
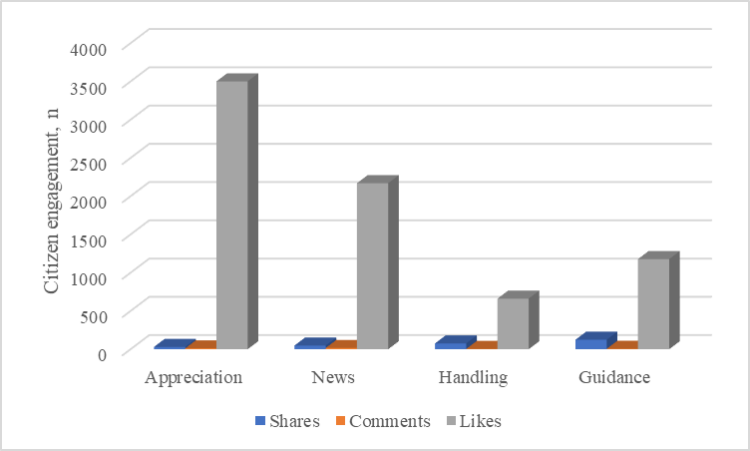
Average amount of citizen engagement in the form of shares, likes, and comments, grouped by content type.

The 355 collected short videos had large variations in the level of citizen engagement. Overall, 32.1% of videos (n=114) had less than 10 shares, while 9.9% of videos (n=35) were shared more than 100 times. The gap in the number of likes was also significant, with 56 videos (15.8%) receiving less than 100 likes and 10 videos (2.8%) receiving more than 10,000 likes. For comments, 8 videos received more than 50 comments, while 18.9% of videos (n=67) did not receive any comments. Among all videos, 154 (43.4%) were related to guidance for clinicians, patients, and ordinary citizens, followed by information concerning the government’s handling of the pandemic (n=100, 28.2%), and the latest news about COVID-19 (n=61, 17.2%). Posts about appreciation toward frontline emergency services only represented about 11.3% (n=40) of posts. On average, the appreciation posts received the highest number of likes (mean 3500.28 [SD 8086.11]), while guidance and government handling information received the least (mean 660.97 [SD 1493.57] and mean 1177.60 [SD 3952.23], respectively; Figure 2). Moreover, videos related to guidance information experienced the highest level of sharing (mean 124.12 [SD 624.95]), while videos pertaining to the latest news had the highest number of comments (mean 16.07 [SD 21.44]). Of the other characteristics of videos, the length of all videos collected ranged from 11 seconds to 493 seconds (mean 96.53 [SD 81.78]). Furthermore, the average length of video titles was 34.75 characters (SD 6.79; range 15-54). For dialogic loop, 203 videos (57.2%) either raised or answered questions to deliver information. Further, the emotional valence of video titles was positive on average (mean 0.71 [SD 0.30]).

### Hypothesis Testing

Table 2 shows estimates of the negative binomial regression models that predicted the number of likes, shares, and comments for each coded short video uploaded during the COVID-19 pandemic. Hypothesis 1 posited that longer videos were less likely to attract citizen engagement. Models 2 and 3 in Table 2 show that video length has a negative and statistically significant association with the number of likes (incidence rate ratio [IRR]=0.19, *P*<.001) and comments (IRR=0.39, *P*<.001) received. The IRR value shows that a one-unit increase in video length leads to a decrease in the number of likes and comments by a factor of 0.19 and 0.39, respectively. However, the relationship between video length and number of shares is not significant. Thus, hypothesis 1 was partially supported.

**Table 2:** Predicting citizen engagement with government social media.

Variables		Shares (Model 1)		Likes (Model 2)		Comments (Model 3)	
		Incidence rate ratio	*P* value	Incidence rate ratio	*P* value	Incidence rate ratio	*P* value
Video length		0.81	.59	0.19	<.001	0.39	<.001
Title length		24.25	.01	8.50	.03	7.85	.02
Dialogic loop		0.56	.03	0.67	.07	0.68	.07
**Content type (reference: appreciation)**							
	News	1.15	.67	0.46	.004	0.99	.96
	Handling	5.16	<.001	0.97	.94	1.06	.86
	Guidelines	7.31	<.001	0.76	.36	.89	.67
Constant		0.28	.51	1398.47	<.001	1.85	.68
Log likelihood		–1797.08	N/A^a^	–2816.56	N/A	–1026.53	N/A
Pseudo *R*^2^ (%)		1.41	N/A	1.67	N/A	3.68	N/A

^a^N/A: not applicable.

Hypothesis 2 proposed that a longer video title increases the level of citizen engagement. According to Table 2, title length indeed plays an important role in predicting the number of shares (IRR=24.25, *P*=.01), likes (IRR=8.50, *P*=.03), and comments (IRR=7.85, *P*=.02). A one-unit increase in title length can result in a 2325% increase in the number of shares, a 750% increase in the number of likes, and a 685% increase in the number of comments. Thus, hypothesis 2 was supported.

Hypothesis 3 proposed that videos with multiple dialogic features were more likely to attract citizen engagement. Model 1 in Table 2 shows that dialogic loop is negatively associated with the number of shares (IRR=0.56, *P*=.03). This means that dialogic loop decreases willingness to share by about 44%. In addition, Models 2 and 3 show that dialogic loop is not associated with the number of likes and comments received. Thus, hypothesis 3 was not supported.

Hypothesis 4 proposed that the degree of citizen engagement was influenced by the content shown in the videos. Since content type is a categorical variable, we treated appreciative posts as the reference group. Model 1 in Table 2 shows that government handling information (IRR=5.16, *P*<.001) and guidelines information (IRR=7.31, *P*<.001) were positively correlated with the number of shares. The IRR values mean that, compared to appreciative information, handling information would result in a 416% increase in the number of shares, while guideline information would lead to a 631% increase. Videos about the latest news were found to be negatively related to the number of likes (IRR=0.46, *P*=.004), which means that posts about the latest news would result in a 54% decrease in the number of likes received, compared to posts about appreciative information. Interestingly, the number of comments did not vary significantly across different types of video content. Thus, hypothesis 4 was partially supported.

**Figure 3:**
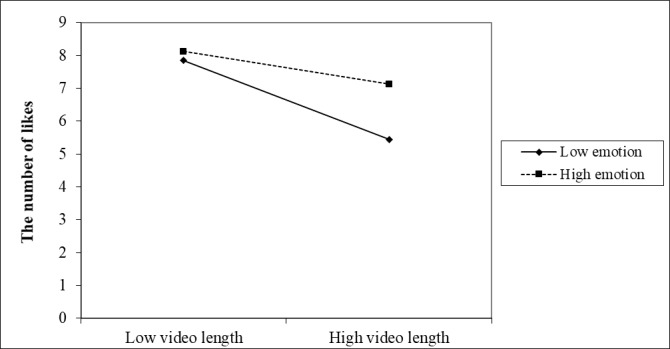
Interaction effects of video length and emotion on the number of likes received.

**Figure 4:**
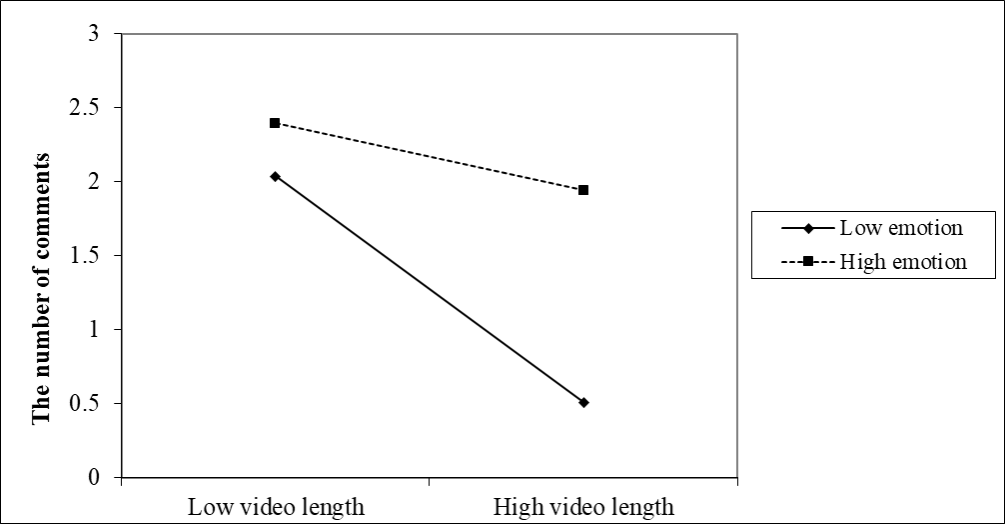
Interaction effects of video length and emotion on the number of comments received.

We investigated the conditional impacts of the predictors by entering emotional valence and interaction variables into the negative binomial regression model. As seen in Table 3, the relationship between video length and citizen engagement is moderated by the emotional valence of a video’s title. The interaction between video length and emotion is positively related to the number of likes (IRR=21.72, *P*=.04) and comments (IRR=10.14, *P*=.047; see Models 2 and 3). As Figure 3 shows, the gap in the number of likes between videos with positive emotion and negative emotion is only significant when the video length is long. The more negative the emotion in the video’s title, the lower the number of likes received. This pattern is almost the same for the number of comments (Figure 4). The lowest number of comments occurred when the video was long and had negative emotion. Emotion also moderates the relationship between content type and the number of shares. As shown in Table 3 (Model 2), for guidelines (IRR=7.59, *P*=.04) and government handling (IRR=14.48, *P*=.04) information, the more positive emotion a video’s title had, the higher the number of shares the video received, in comparison to appreciative news. Videos related to the latest news (IRR=66.69, *P*=.04) received more likes when their titles had higher levels of positive emotion. Interestingly, the title’s emotional valence has a weak effect or no effect on the relationship between content and other types of engagement behavior. Overall, emotion plays a moderate role in the relationship between video length, content type, and citizen engagement, but patterns vary across different types of engagement.

**Table 3:** The moderating role of emotional valence in predicting citizen engagement.

Variables			Shares (Model 1)		Likes (Model 2)		Comments (Model 3)	
			Incidence rate ratio	*P* value	Incidence rate ratio	*P* value	Incidence rate ratio	*P* value
Video length			0.67	.28	0.12	<.001	0.28	<.001
Title length			32.48	.008	7.84	.08	3.69	.23
Dialogic loop			0.68	.15	0.70	.13	0.63	.03
**Content type (reference: appreciation)**								
	News		2.36	.25	0.17	.02	2.03	.23
	Handling		0.51	.49	2.03	.55	0.99	.99
	Guidelines		1.15	.86	1.63	.65	3.02	.17
**Interaction**								
	Emotion		1.10	.91	5.70	.05	4.50	.03
	Emotion × video		0.45	.56	21.72	.04	10.14	.047
	Emotion × title		1.60	.91	4.41	.77	0.60	.90
	**Emotion × content type (reference: appreciation)**							
		News	0.11	.19	66.69	.04	0.09	.06
		Handling	14.48	.04	0.38	.50	0.98	.99
		Guidelines	7.59	.04	0.37	.43	0.21	.10
Constant			0.25	.51	3313.61	<.001	9.84	.22
Log likelihood			–1785.57	N/A^a^	–2806.04	N/A	–1017.43	N/A
Pseudo *R*^2^ (%)			2.04	N/A	2.03	N/A	4.53	N/A

^a^N/A: not applicable.

## Discussion

### Principal Findings

This pioneering study is the first to attempt to reveal how to promote citizen engagement with official government TikTok accounts during the COVID-19 crisis. To reveal the specific mechanisms of engagement, we proposed a moderated model, which investigated the moderating role of emotional valence on the effects of video length, title length, dialogic loop, and content type on the three dimensions of citizen engagement with the official TikTok account of the NHCC.

First, we found that shorter videos and videos with longer titles generate greater engagement in general. On the one hand, video length negatively and significantly influenced the number of likes and comments received. This conclusion confirms the findings of Zhu et al [19], who found that citizens prefer shorter TikTok videos. We extend their findings in the context of public health crises. Shorter videos can better satisfy public demands for timely consumption and relaxation during fragmented time and promote the public’s continuous use of TikTok videos, including increasing sharing, liking, and commenting behaviors, thereby improving engagement [1,26]. On the other hand, videos with longer titles generate greater levels of sharing, liking, and commenting, simultaneously. This is consistent with the findings of Halvey and Keane [23], who established that YouTube videos with longer titles receive more views. Longer video titles help to enhance the logical and interactive elements of expression but can also deliver more information. In contrast, shorter titles may compromise the integrity and accuracy of video content cues. Hence, the longer the video title is, the more accurately and effectively the core content can be presented, which increases the likelihood of receiving likes, shares, and comments. However, this conflicts with the result of a recent study on YouTube marketing, in which video titles with a lot of information were associated with a lower number of video views [36]. This discrepancy may be due to the evolving functionalities of YouTube, the increasing large volume of available videos, and the changing demographics of YouTube users. With the surge of new videos, the younger generation’s attention is precious and they are more likely to be attracted by videos with shorter titles. It remains to be seen whether this change also applies to TikTok.

Second, this study found that content related to the government’s handling and guidance for stakeholders promotes citizens’ reposting behavior, while information related to the latest news about the crisis reduces the number of likes received. This further expands on the conclusion of Chen et al [1], who found that posts related to the government’s handling of information (released by the official Sina Weibo account of the NHCC) promoted citizen engagement on the TikTok platform. This suggests that, whether it is a general social networking platform or one focused on video sharing, the public are concerned about the government’s handling of the crisis, which increases the level of citizen engagement. However, compared with Sina Weibo users, content related to the latest news about the COVID-19 crisis has very limited attraction for TikTok users. This may be because short videos that are related to the latest news about the COVID-19 crisis are mostly a combination of plain text and pictures, which are rougher and less interactive.

Interestingly, this study discovered that higher levels of dialogic loop significantly reduce the number of shares received. This conflicts with existing studies that examine how dialogic loops affect citizen engagement [1,25,37]. It is worth noting that previous studies that support the positive impact of dialogic loop on citizen engagement were all concentrated on nonvideo social media platforms, such as Facebook, Twitter, and Sina Weibo, while this study focused on TikTok, a short video-sharing platform. This indicates that the effect of dialogic loop on citizen engagement may vary depending on the type of social media platform. This study measured dialogic loop by video title. Although a video’s title is a concentrated expression of the content displayed, it is not completely equivalent to the interactivity of the video itself. In addition, this inconsistency may also be due to different measurements of dialogic loop. In fact, researchers have not reached a consensus on how to evaluate dialogic loop on social media platforms [1]. For example, Men et al [37] measured the dialogic loop of chief executive officers’ Facebook accounts from three dimensions, namely replying to comments, liking user comments, and launching surveys. Chen et al [1] adopted 5 dimensions, including using hashtags and mentions, launching surveys, asking users questions, and answering users’ questions, to assess the dialogic loop of government social media. Both studies found that dialogic loop is positively associated with social media engagement. A study by Wang and Yang [35] used questions and answers published by nonprofit and for-profit organizations on Twitter to measure dialogic loop. Interestingly, our findings are comparable in that responding to questions resulted in the lowest numbers of likes and retweets, while asking questions had little effect on the number of likes and shares received [35].

Most importantly, this study confirmed that emotional valence can moderate the impact of video length and content type on citizen engagement, although the moderating effects vary in the three dimensions of citizen engagement. For longer videos, the more negative emotion the video’s title displays, the lower the number of likes that the video would receive. This pattern is almost the same for the number of comments. This means that longer videos with positive titles receive higher numbers of likes and comments. For short videos related to the government’s handling of the pandemic and guidance for stakeholders, positive titles received more shares. Videos about the latest news received more likes if the title had a higher level of positive emotion. This demonstrates the important role of the emotional valence of video titles in promoting citizen engagement through government TikTok accounts. This can be explained by The Social Sharing of Emotion Theory (SSET), which was proposed by Rimé and colleagues in the 1990s. The SSET posits that emotion is a critical motivator for information sharing and social interaction. In our daily life, emotional events can happen anywhere and at any time, and those who experience emotional events are likely to talk about them and the feelings experienced [38]. Through this social sharing of emotion, individuals can express their emotional state and facilitate the establishment of interpersonal relationships [38]. Considering the existence of positivity bias, information with positive emotion creates a good experience for the receiver, and builds a positive image of the information sender [39]. Compared to negative emotion, positive emotion can activate individuals’ desire to seek and share information [40]. Therefore, when individuals encounter emotional content from the title of a TikTok video, they will have an urgent need to share this emotional experience. Further, positive emotion will strengthen this behavior, facilitating public engagement on TikTok, enhancing likes, shares, and comments.

### Contribution and Implications

This research makes the following contributions. First, we not only empirically examined how governments have used new video-based social media platforms for public engagement in the context of a public health crisis, but also built a model with emotional valence as a moderator to explain the underlying mechanism of how video length, title length, dialogic loop, and content type affects public engagement with government TikTok accounts. Second, we reveal that although shorter videos are likely to attract more likes and comments, for longer videos, the emotional valence of the title is of critical importance. For long videos, the more positive the title, the more likes and comments it receives, and vice versa. Third, our study found that emotional valence displayed in the title plays a critical role in the impact of content type on public engagement. For videos focused on guidance and the government’s handling of the crisis, the more positive the emotion displayed in the video title, the higher the number of shares it received. In addition, videos related to the latest news received more likes when the title had higher levels of positive emotion. Fourth, our study found that dialogic loop is likely to reduce certain types of public engagement.

This study has several practical implications. First, TikTok has become an indispensable channel for government-citizen communication, and governments should actively embrace this new method of communication. Second, for government entities producing videos for TikTok, shorter video length is preferred. If the video length is relatively long, it is better to have a title with positive emotion rather than negative emotion. Third, after the public health crisis, governments are advised to publish more information about their handling of the crisis, provide further guidance for stakeholders, and upload videos with positive emotion in the title to facilitate delivery.

### Limitations and Future Recommendations

This study has several limitations. First, we focused on the official TikTok account of the NHCC. Whether the results of this research are equally applicable to local health departments is worthy of investigation. Second, it is worth exploring if the conclusions are similar in Western countries. There are notable differences between TikTok and YouTube [41]; although both of them are video-based social networking platforms, TikTok videos are much shorter than those on YouTube, and there are various easy-to-use templates. The typical length of a TikTok video may be just 30-40 seconds, while YouTube videos can be more than 15 minutes long. In addition, TikTok is a mobile-only social media app, while YouTube operates via both a website and mobile app. Their functionalities also differ; for example, YouTube has a dislike button, while TikTok does not. Furthermore, compared with the individualism advocated by Western countries, China emphasizes collectivism. Moreover, the number of comments found by our study could be influenced by the presence of censorship in China. Third, due to the constraints of the content analysis methods, some important variables were not employed in our research model (eg, camera view). Wang [42] found that the camera view of short videos on TikTok significantly affects the audience’s immersion and social presence, which are important elements in promoting citizen engagement. We attempted to use content analysis to determine the type of camera view but it was difficult for the encoders to reach consensus and the interreliability was relatively low. Fourth, the significant negative impact of dialogic loop on reposting behavior deserves further investigation, although we infer that this may be due to differences between TikTok and other non-video–related social media platforms. In addition, our study did not obtain information from NHCC regarding their video and post creation process. Future research could use a qualitative approach to find out how the government TikTok operates in terms of content selection, rules, and strategies during a health crisis. Data from TikTok users could also enrich future studies [43,44].

### Conclusions

It is evident that fostering engagement through video sharing on TikTok provides public health departments with the ability to promote citizen engagement and accelerate the dissemination of health information. Nonetheless, how to sustain this positive effect is still unclear. Through analysis of 355 COVID-19–related TikTok videos scraped from the official NHCC account, this study addresses this knowledge gap by considering emotional valence as a moderator that determines the effect that video length, title length, dialogic loop, and content type have on the three dimensions of citizen engagement. In general, shorter videos and longer video titles elicit greater citizen engagement. Citizens prefer to view content about the government’s handling of the pandemic and guidance for stakeholders, and actively repost these TikTok videos. We confirmed the important role of emotional valence as a moderator and discovered the specific moderating mechanisms. Longer videos with positive titles received higher numbers of likes and comments. For short videos related to government handling and guidance for stakeholders, positive titles received more shares. Videos related to the latest news received more likes when the title had a higher level of positive emotion. Public health departments should create shorter videos with longer titles, with content focused on the government’s handling of the crisis and guidance for stakeholders. Moreover, in the future, video producers should consider the emotional valence of titles and align it with the content type and video length.

## Abbreviations

CNNIC: China Internet Network Information Center
IRR: Incidence Rate Ratio
NHCC: National Health Commission of China
SSET: Social Sharing of Emotion Theory
